# Metabolome-Wide Reprogramming Modulated by Wnt/β-Catenin Signaling Pathway

**DOI:** 10.4014/jmb.2211.11013

**Published:** 2022-11-18

**Authors:** Soo Jin Park, Joo-Hyun Kim, Sangtaek Oh, Do Yup Lee

**Affiliations:** 1 Department of Agricultural Biotechnology, Seoul National University, Seoul 08826, Republic of Korea; 2 Department of Bio and Fermentation Convergence Technology, Kookmin University, Seoul 02707, Republic of Korea; 3 Department of Interdisciplinary Program for Bio-Health Convergence, Kookmin University, Seoul 02707, Republic of Korea; 4 Interdisciplinary Program in Agricultural Genomics, Seoul National University, Seoul 08826, Republic of Korea; 5 Center for Food and Bioconvergence, Research Institute for Agricultural and Life Sciences, Seoul National University, Seoul 08826, Republic of Korea

**Keywords:** Metabolomics, Wnt/β-catenin signaling, mass spectrometry, fatty acid metabolism

## Abstract

A family of signal transduction pathways known as wingless type (Wnt) signaling pathways is essential to developmental processes like cell division and proliferation. Mutation in Wnt signaling results in a variety of diseases, including cancers of the breast, colon, and skin, metabolic disease, and neurodegenerative disease; thus, the Wnt signaling pathways have been attractive targets for disease treatment. However, the complicatedness and large involveness of the pathway often hampers pinpointing the specific targets of the metabolic process. In our current study, we investigated the differential metabolic regulation by the overexpression of the Wnt signaling pathway in a timely-resolved manner by applying high-throughput and un-targeted metabolite profiling. We have detected and annotated 321 metabolite peaks from a total of 36 human embryonic kidney (HEK) 293 cells using GC-TOF MS and LC-Orbitrap MS. The un-targeted metabolomic analysis identified the radical reprogramming of a range of central carbon/nitrogen metabolism pathways, including glycolysis, TCA cycle, and glutaminolysis, and fatty acid pathways. The investigation, combined with targeted mRNA profiles, elucidated an explicit understanding of activated fatty acid metabolism (β-oxidation and biosynthesis). The findings proposed detailed mechanistic biochemical dynamics in response to Wnt-driven metabolic changes, which may help design precise therapeutic targets for Wnt-related diseases.

## Introduction

A family of signal transduction pathways called wingless-type (Wnt) signaling pathways is implicated in the regulation of developmental events such as cell proliferation, differentiation, and density during embryogenesis [1, 2]. One of the significant role players in the canonical Wnt signaling pathway is β-catenin [3]. Wnt protein binds to low-density lipoprotein receptor-related proteins 5 and 6 (LPR5/6) and frizzled receptor (FZD) complexes, which leads to the accumulation of unphosphorylated β-catenin [4]. T-cell factor/lymphoid-enhancer binding factor (TCF/LEF) and β-catenin interact to regulate the transcription of Wnt-responsive genes [5]. The transcription of Wnt target genes modulates the cell cycle, including *c-MYC*, *CYCLIN D1*, *PDK*, and *AXIN-2*.

Clinical importance has been proposed for Wnt/β-catenin signaling pathway in both normal homeostasis and abnormal status of organs [6]. The dys-regulation of Wnt/β-catenin signaling pathways causes a range of diseases, such as cancer, metabolic disease, cardiovascular disease, and neurodegenerative diseases [5, 7]. The signaling pathway has been extensively studied as therapeutic target for a broad spectrum of diseases [8-11]. Primary approach has been focusing on the discovery of an inhibitor that blocks the Wnt/beta-catenin signaling pathway. However, the clinical application still remains challenged due to the Wnt signaling system's complexity. Thus, a more precise and systematic characterization of a metabolic signature may help better understand Wnt signaling pathway-associated biological events, including disease. Indeed, our current study aims to clarify global metabolic regulation and physiology modulated by Wnt/β-catenin signaling. Un-targeted metabolic profiling was applied on human embryonic kidney 293 cells (HEK 293 cells), which are popular for their ease of growth and transfection. Wnt-3a was used to activate Wnt/β-catenin signaling to investigate the Wnt-specific responsive metabolism in time-responsive manners.

## Materials and Methods

### Cell Cultures and Luciferase Assay

HEK293 cells (ATCC, USA) were cultured in DMEM supplemented with 10% fetal bovine serum (FBS) and 1%penicillin/streptomycin (Pen/Strep). Wnt3a-secreting L cells (ATCC) were utilized for Wnt3a-conditioned medium (Wnt3a-CM). The development of HEK293-firefly luciferase (FL) reporter cells was conducted by transfection of β-catenin/TCF-dependent FL reporter (TOPFlash) as previously described [12]. The Dual-Luciferase Assay Kit (Promega, USA) was used in accordance with the manufacturer's instructions.

### Western Blot Assays

Freeze-dried cell samples (1 mg) were dissolved in 100 μl of lysis buffer. Lysis buffer consisted of protein inhibitor and radioimmunoprecipitation assay (RIPA) buffer. To determine the quantity of protein, Bradford assay was employed. Equal volumes of lysates were separated by SDS-PAGE (Invitrogen, USA) and transported onto nitrocellulose membranes (Bio-Rad Laboratories, USA). The blocking for western blot analysis was performed by soaking in SuperBlock Blocking Buffer (Thermo Fisher Scientific, USA) for one hour. Anti-β-catenin (BD Transduction Laboratories, USA) was utilized for western blot detection of β-catenin. β-actin was chosen as a loading control using anti-actin (Cell Signaling Technology, USA). Blotting was visualized and quantified using the ECL system (Santa Cruz Biotechnology, USA) and the ImageJ software (NIH, USA).

### RNA Isolation and RT^2^ qPCR Array Analysis

Total RNA was isolated using RNeasy mini kit (Qiagen, Germany) and the techniques in the associated protocol. cDNA was synthesized with the RT2 First Strand kit (Qiagen, Germany). cDNA was mixed with RT2 qPCR master mix for performance of SYBR Green-based real-time PCR [13]. The CFX96 real time PCR detection system (Bio-Rad) was utilized for Real-time PCR. The information of custom qPCR array is shown in Table S1.

### Metabolite Extraction

Freeze-dried cell samples were disrupted using a Mixer Mill MM400 (Retsch GmbH, Germany) with a single steel bead (frequency 28 Hz, time 30 sec). A 750 μl of extraction solvent (isopropyl alcohol:methanol:water, 3:3:2, v/v/v) was used to extract the metabolites of the powder samples. The samples were homogenized in Mixer Mill MM400 at frequency of 25 Hz for 10 sec, followed by sonication (5 min) and centrifugation (5 min, 13200 ×*g* at 4°C).

### GC-TOF MS Analysis

N-methyl-N-trimethylsilyltrifluoroacetamide (MSTFA) derivatization was performed in two steps [14, 15]. First, samples were methoxyaminated using methoxyamine hydrochloride (Sigma-Aldrich, USA) in pyridine. Second, the trimethylsilylation step used MSTFA (MSTFA + 1% trimethylsilyl chloride; Thermo Fisher Scientific). For the retention time index, a mixture of fatty acid methyl esters [FAMEs] was added. The retention index marker contained 13 FAMEs. GC systems was an Agilent 7890B gas chromatograph system (Agilent Technologies, USA) with an RTX-5Sil MS column (Restek, Gellefonte, PA). The MS system was Leco Pegasus HT time-of-flight mass spectrometer controlled using the ChromaTOF software (LECO, St. USA). The mass spectrum was acquired in the mass range of 85-500 *m/z* at a rate of 20 spectra/s. The data files were processed by the Binbase algorithm with Fiehn library [16].

### Liquid Chromatography (LC)-Orbitrap Mass Spectrometry (MS) Analysis

Details of the metabolic profiling methods can be found in our previous publication [17]. In summary, chromatographic analysis was carried out using an Ultimate-3000 Ultra Performance Liquid Chromatography (UPLC) system (Thermo Fisher Scientific). Dried extracts were suspended with distilled water (DW) and acetonitrile (ACN) (70:30, v/v) solution. The samples were separated in 150 × 2.1 mm UPLC BEH 1.7-μm hydrophilic interaction liquid chromatography (HILIC) column (Waters, USA) equipped with VanGuard pre-column (Waters). The mobile phases were solution A as DW: ACN (98:5, v/v) with 68 mM ammonium hydroxide and 19 mM ammonium acetate and solution B as ACN. Mass spectrometry was performed using Q-Exactive Plus instrument (Thermo Fisher Scientific). MS scan mode was Full-MS coupled with data-dependent MS/MS (Full-ddMS2) scan. Scan mass range was 85 to 1275 m/z. The data processing was performed with MS-DIAL, including peak alignment and metabolite identification. (Precursor ion *m/z* tolerance, 0.005 Da; Product ion *m/z* tolerance, 0.05 Da, MS/MS similarity score: 70%)

### Statistical Analysis

Statistics were applied to all quantitative data value collected from gas chromatography coupled to time-of-flight mass spectrometry (GC-TOF MS) and LC-Orbitrap MS. Missing value imputation using random forest, univariate statistics, PERMANOVA, chemical classification based on Mesh, chemical similarity enrichment analysis (ChemRICH) [18] were performed using R (https://github.com/CHKim5/LMSstat). Principle component analysis (PCA), partial least squares-discriminant analysis (PLS-DA) were performed using SIMCA 17 software (Umetrics AB, Sweden). Significance analysis of microarrays (SAM) and hierarchical clustering heat map were performed using MetaboAnalyst 4.0 (http://metaboanalyst.ca). The metabolite-protein interaction network was constructed using STITCH (http://stitch.embl.de) [19]. Prism 8 (GraphPad Software, USA) was used for box & whisker plots. The figures were created and reshaped by Adobe Illustrator (Adobe Systems Inc., USA) and BioRender.com.

## Results and Discussion

### Activation of the Wnt/β-Catenin Pathway by Wnt3a-CM in HEK293 Cells

**Fig. 1:**
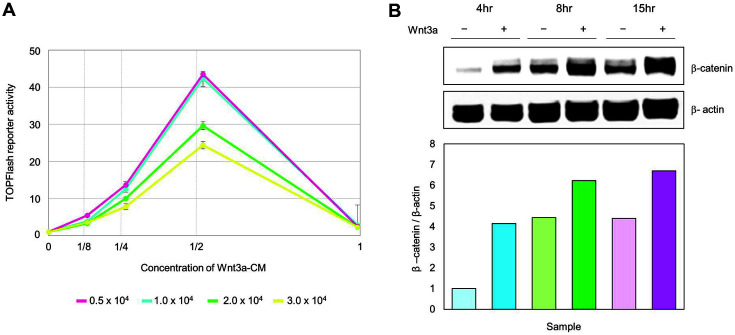
Up-regulated β-catenin in HEK293 cells by Wnt3a-CM. (**A**) Optimization of Wnt3a-CM concentration for treatment. β-catenin response transcription activities were measured using luciferase assay at 15 h. (**B**) The strongest upregulation of β-catenin protein was determined at 15 h. The level of β-catenin expression was quantified by western blot.

First, we determined the optimal treatment concentration of the Wnt3a-CM. Four different concentrations of Wnt3a-CM were prepared; 100%, 50%, 25%, and 12.5%, diluted with corresponding volume of normal culture medium. We measured β-catenin response transcription (CRT) activity based on luciferase assay using HEK293-FL reporter cells previously established [12]. HEK293 FL reporter cells with four different seeding cell numbers (5e3, 1e4, 2e4, and 3e4) were incubated with Wnt3a-CM for 15 h. The TOPFlash reporter activity was gradually increased according to Wnt3a-CM concentration, but rather decreased in 100% Wnt3a-CM (Fig. 1A). Accordingly, 50% of Wnt3a-CM was selected as the treatment concentration for metabolic profiling.

HEK293 cells were treated with 50% Wnt3a-CM and harvested at 4 h, 8 h, and 15 h to profile the time-dependent metabolic change. The cytosolic protein level of β-catenin was determined based on western blot analysis to examine the impact of Wnt3a on Wnt/β-catenin signaling. The results indicated time-dependent induction of β-catenin by Wnt3a-CM treatment (Fig. 1B). The amount of intracellular β-catenin was at the highest level at 15 h-treatment. The Wnt3a-CM treatment activates Wnt/β-catenin pathway by induction of β-catenin [20-22].

### Unique Metabolic Phenotype Triggered by Wnt3a Treatment in a Timely Resolved Manner

We performed mass spectrometry-based integrative metabolic profiling of HEK293 cells using GC-TOF MS and LC-Orbitrap MS. A total of 321 metabolites were annotated based on the combined MS analysis. The metabolites were classified as carboxylic acids and derivatives (24%), organooxygen compounds (12%), fatty acyls (10%), organonitrogen compounds (5%), and miscellaneous compounds. For statistical analysis, the data were combined, following total ion count (TIC) normalization and unit variance (UV) scaling with mean centering.

**Fig. 2:**
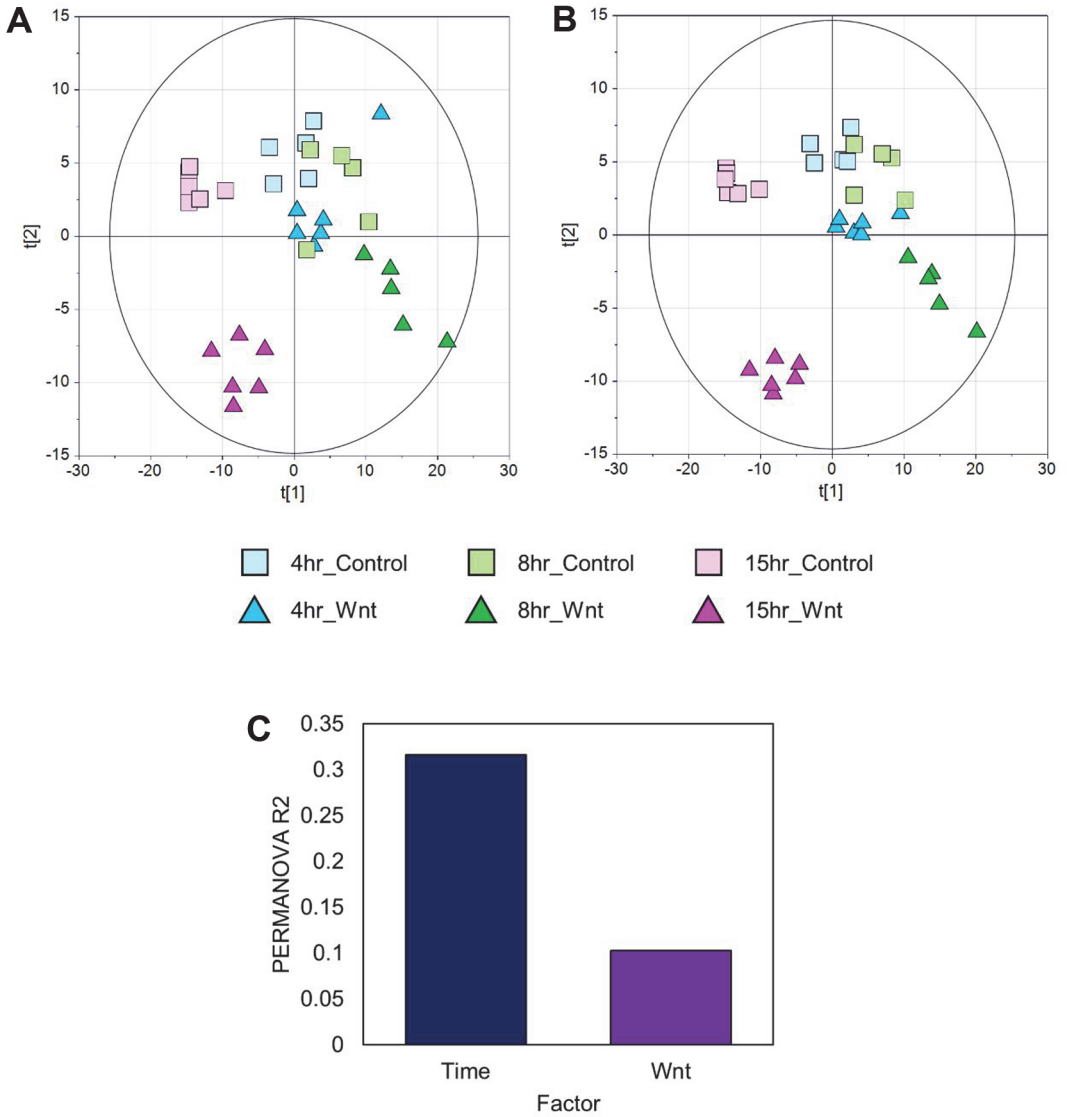
Differential metabolic modulation varying by time and treatment activated Wnt/β-catenin signaling. (**A**) PCA and (**B**) PLS-DA score scatter plot of the HEK293 metabolome. (**C**) Explained variance (%) of the time and Wnt treatment calculated by PERMANOVA.

First, unsupervised multivariate statistics was performed to overview the metabolic phenotype according to Wnt3a treatment. PCA score scatter plot showed that the metabolic phenotype was distinguished by Wnt3a treatment according to the different time points (Fig. 2A). The discrimination by component 1 (30.1%) and component 2 (10.1%) was associated with culture time and Wnt3a treatment, respectively. PLS-DA, supervised multivariate statistics, confirmed the discriminant profiles (R2Y=0.982 and Q2=0.829) (Fig. 2B). The reliability of model was validated using ANOVA of cross-validated predictive residuals (CV-ANOVA), which demonstrated a suitable model power (the significance level *p*-value = 0.01).

Further, permutational multivariate analysis of variance (PERMANOVA) was used to quantitatively evaluate the effect of factors on metabolome profiles. Treatment time showed the highest explained variance (R2 = 0.316, *p*-value = 0.001) (Fig. 2C). Wnt3a treatment was also significant factor explaining the variation of metabolome (R2 = 0.103, *p*-value = 0.001). The results suggested that the metabolome was interactively modulated by the combination of Wnt3a treatment and treatment time.

### Wnt3a Treatment-Specific Metabolic Response in Hexoses and Fatty Acids

We further identified the metabolites specific to Wnt3a treatment based on univariate statistics, Mann-Whitney U test. The significant differences were detected in 38, 64, and 105 metabolites at 4 h, 8 h, and 15 h, respectively (*p*-value < 0.05) (Table S2). The number of significantly altered metabolites gradually increased in a time-dependent manner. Thirteen metabolites showed common significant alteration by Wnt3a treatment at the all-time point (Fig. S1A). Glucose 1-phosphate and ergosterol were significantly up-regulated by Wnt3a treatment, whereas 3-indoleacetic acid, glutamine, aminoadipate, N-acetyl putrescine, galactose, glucose, oxoproline, and succinic acid were significantly down-regulated (Fig. S1A). The down-regulation of glucose, succinic acid, and glutamine may imply the increased flux to glycolysis, the tricarboxylic acid (TCA) cycle and glutaminolysis. Wnt3a-induced β-catenin increase has been reported for the association with cell proliferation [23, 24], which leads to the metabolic change by hypoxic mechanism [25, 26]. It has been proposed that proliferating cells utilize glycolysis, the tricarboxylic acid (TCA) cycle, and glutaminolysis for energy production [25].

**Fig. 3:**
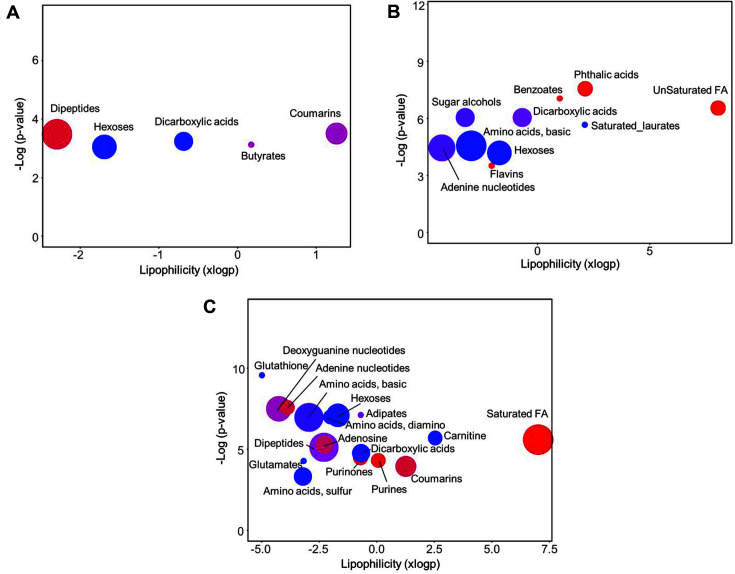
Wnt/β-catenin signaling-specific response by time point at the chemical class levels. Chemical enrichment analysis using significantly altered metabolites (Mann-Whitney U test, *p*-value < 0.05) according to the Wnt at 4 h (**A**), 8 h (**B**), and 15 h (**C**). Circle size means the number of the metabolites in class. Increased and decreased metabolites in Wnt group are shown by the colors red and blue, respectively. Purple color indicates the presence of both increased and decreased metabolites.

**Fig. 4:**
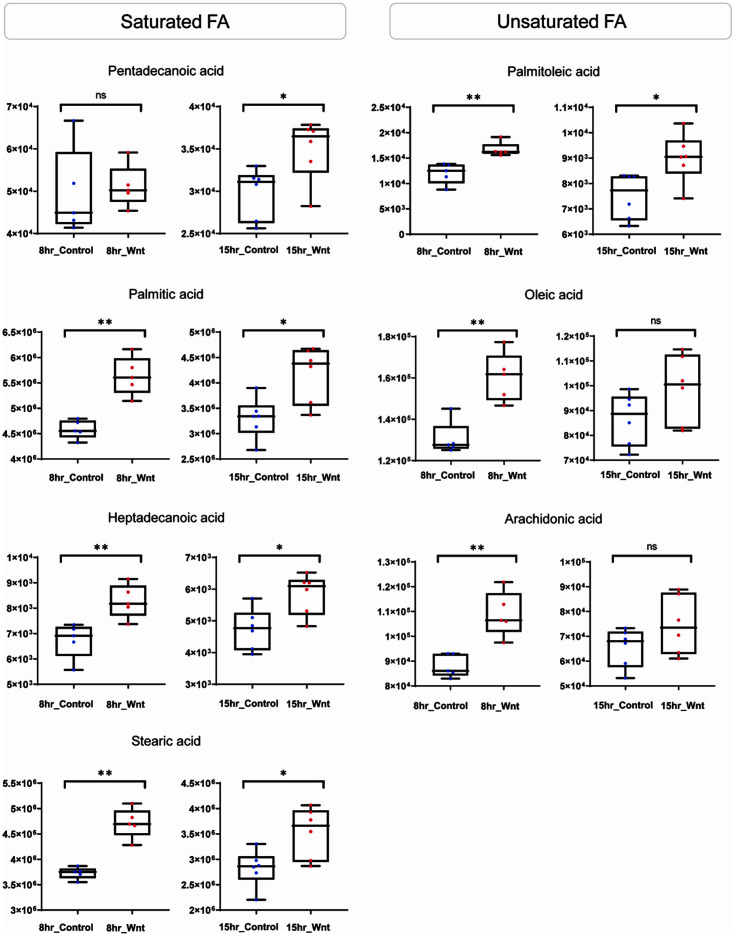
Relative abundances of statistically changed fatty acid in 8 h and 15 h-Wnt3a treatment cells. Box & Whisker plot of significantly up-regulated metabolites in 8 h- and 15 h-Wnt treatment cells. *: Mann-Whitney U test, *p*-value < 0.05, **: *p*-value < 0.01, ns: no-significance.

To systematically characterize the metabolic modulation at the level of chemical class, chemical similarity enrichment analysis (ChemRICH) was performed for each time point. Quantitative evaluation was done based on statistical significance (Mann-Whitney U test, *p*-value < 0.05) and fold change (Wnt3a group / control group). Among significantly altered chemical classes, hexoses showed lower abundances in the Wnt3a group compared to normal group at all time points (Fig. 3). The down-regulation was the most distinctive change at 15 h, which was shown in fructose, galactose, glucose, mannose, and tagatose. Basic amino acids showed significantly lower contents in Wnt3a group, particularly at 8 h and 15 h (Figs. 3B and 3C). Three amino acids and 5 amino acids were decreased at 8 h and 15 h, respectively. The synchronized alteration of amino acid was at maximal level at 15 h, which included glutamine, arginine, beta-homolysine, lysine, and asparagine. The significant alteration of dicarboxylic acids by Wnt3a was also determined at 8 h and 15 h. Unsaturated fatty acids were a distinct metabolic feature specific to 8 h-treatment, in which palmitoleic acid (C16:1), oleic acid (C18:1), and arachidonic acid (C20:4) were significantly up-regulated (Figs. 3B and 4). The enrichment of saturated fatty acid was predominant at 15 h. The fatty acids were pentadecanoic acid (C15:0), palmitic acid (C16:0), heptadecanoic acid (C17:0), and stearic acid (C18:0) (Figs. 3C and 4). In addition, FA C16:0, C17:0, and C18:0 were significantly increased in the Wnt3a group at 8 h.

### Activation of Fatty Acid Metabolism by Wnt3a at Gene Expression Level

To causatively determine the up-regulation of fatty acids by Wnt3a, RT-qPCR analysis was conducted on the samples with 15 h treatment, which showed the most differential metabolic regulation. The customized PCR array included 85 genes, mapped to fatty acid metabolism, including biosynthesis, transport, ketogenesis, and triacylglycerol synthesis.

The quality of the PCR data was verified for reverse transcription (RT) efficiency, genomic DNA contamination, and PCR array reproducibility. The threshold cycle (Ct) values of positive PCR control (PPC) were 20.85 to 21.30, indicating reliable technical reproducibility. The delta value of the reverse transcription control (RTC) mean and PPC mean was 4.09, which demonstrated reverse transcription PCR was not affected by sample impurities. The genomic DNA control (GDC) Ct values of all samples were above 35, showing no genomic DNA contamination. All Ct values of genes were normalized with housekeeping genes (*GAPDH*, *ACTIN*). Two genes with missing Ct value were excluded from the following data analysis.

**Fig. 5:**
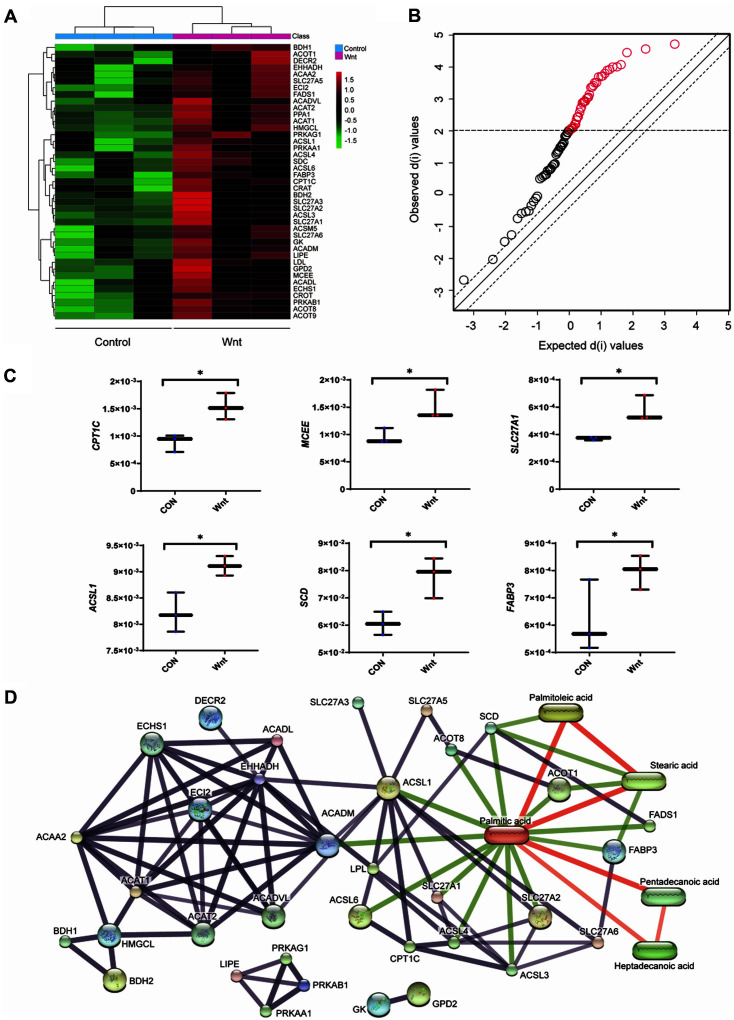
Up-regulation of gene expression associated with fatty acid metabolism by Wnt/β-catenin signaling at 15 h. (**A**) Hierarchical clustering heat map of genes using Euclidean distance (**B**) Significantly up-regulated fatty acid-related genes according to the Wnt/β-catenin signaling based on Significance analysis of microarray (SAM) analysis. The red circle indicates significantly up-regulated ones with the treatment of Wnt3a (Expected score < Observed score) (**C**) Box & Whisker plot of genes which showed an increase with statistical significance according to the Wnt signaling. *: SAM, *q*-value < 0.05. (**D**) An interactive network of protein-metabolite. The oval node indicates seeding metabolites, and the circle node presents protein based on the gene list. An edge indicates a functional link (experimental/biochemical data, association in a curated database, multiple statements in references, or direct/predicted interaction). Edge thickness is the level of confidence. The Red line presents the interaction between metabolites and green edge shows protein-metabolite interaction, and the gray line shows protein-protein interaction.

Heatmap using hierarchical clustering analysis showed differential gene expression level according to Wnt3a treatment. Majority of the target genes were up-regulated by Wnt3a (Fig. 5A). Significance analysis of microarray (SAM) was performed to validate the statistical significance. A total of 41 genes (50 % of all genes) showed differential gene expression between the Wnt3a treatment group and the no-treatment group (*q*-value < 0.05)(Fig. 5B). The highest level of up-regulation was detected in *CPT1C*, which encoded carnitine palmitoyl transferase 1C (Fig. 5C). The CPT1C enzyme catalyzes the carnitinylation of fatty acids for mitochondrial translocation and regulates the oxidation of fatty acids [27]. The second most dynamic up-regulation was determined in MCEE, methylmalonyl-CoA epimerase, involved in the degradation of odd-chain fatty acids (Fig. 5C) [28]. The third highly up-regulated gene, SLC27A1, is acyl-CoA esterification from long-chain fatty acid, which has been reported for an increasing rate of fatty acid oxidation (Fig. 5C) [29].

The integrative information on interactions between small molecules and proteins plays an essential role for elucidating metabolic regulation, signal transduction, and potential drug development [19]. Accordingly, we systematically linked the key metabolites, 6 fatty acids (Mann-Whitney U test, *p*-value < 0.05) to the 41 genes (SAM, *q*-value < 0.05) based on *in-silico* chemical-protein association. The association included experimental evidence of direct chemical-protein interaction and enzyme-substrate relatedness from metabolic pathway, which is all deposited and extractable from STITCH (‘search tool for interactions of chemicals’). The baited proteins and metabolites without interactions were excluded from network. The resultant network identified palmitic acid as a primary hub, which has 16 interactions including 4 metabolites and 8 genes (Fig. 5D). The subsequent over-representation enrichment analysis (ORA) demonstrated key functional modules. The proteins baited by the gene unique to Wnt pertained to fatty acid ligase activity and long-chain & very long-chain fatty acid-CoA ligase activity. *ACSL1* (Acyl-CoA synthetase long-chain family member 1) showed the highest connectivity in the network. *ASCL1* is one of the members associated with fatty acid ligase activity and long-chain fatty acid-CoA ligase activity. ACSL1, long-chain acyl-CoA synthases, synthesizes fatty acyl-CoAs from long-chain fatty acid and accelerates the early stage of fatty acid metabolism [30]. The overexpression of ACSL1 enhances the biosynthesis of triglycerides and β-oxidation, which may meet the requirement for higher proliferation in Wnt-activated cells (Fig. 5C). The activation of ACSL1 was accompanied by up-regulation of other fatty acid-related enzymes, including stearoyl-CoA desaturase (SCD), fatty acid-binding protein (FABP), and carnitine palmitoyl transferase (CPT) [31]. The fatty acyl-CoAs produced by ACSL1 are used to synthesize monounsaturated fatty acids (MUFAs) by SCD [32]. Coherently, our data showed that *SCD1* was up-regulated by Wnt3a with an elevation of the substrate palmitoleic acid (C16:1) and by-product palmitic acid (C16:0) at 15 h (Figs. 4 and 5C). The differential regulation of *SCD1* may be a direct consequence of the activated Wnt/β-catenin signaling pathway, due to the *SCD1* being the target gene of β-catenin [33]. Fatty acid-binding protein 3 (FABP3) transports fatty acids to specific compartment of cell, which regulates lipid droplet generation and lipogenesis [34, 35]. The direct association between fatty acid (*e.g.*, palmitic acid) and *FABP3* has been reported for enhanced de novo synthesis of triglycerides (Fig. 5C) [32]. *CPT1C* was present at elevated levels in our experimental setting, which may involve the activated physiology of mitochondria, including β-oxidation [30].

In our current study, given the activation of Wnt/β-catenin signaling by Wnt3a-CM, we identified the treatment-specific alteration of the metabolome with high temporal resolution. Of note, hexose, including glucose and fructose, showed down-regulation in cell with activated Wnt/β-catenin signaling, which may be associated with promotion of cell proliferation by β-catenin. Cells in proliferation state require the primary carbon source for building blocks and energy production. Our results supported the metabolic evidence from coordinately regulated central carbon/nitrogen metabolism, including glycolysis, TCA cycle, and glutaminolysis. Of note, the activation of fatty acid metabolism was determined at both metabolite and gene levels. The hyper-activity may meet the need for increased energy production via β-oxidation in CRT-positive cells [36]. At the same time, the up-regulation of genes associated with fatty acid biosynthesis may supply lipid building blocks essential for proliferating cells [37]. In summary, metabolic re-programming by Wnt/β-catenin signaling was best featured by the activation of fatty acid metabolism (β-oxidation and biosynthesis). The simultaneous up-regulation entails the dual requirement for actively proliferating cellular environment. Our current study may provide insight into key metabolic regulation linked to Wnt signaling pathway and suggests a new therapeutic target for Wnt-related diseases.

## Supplemental Materials

Supplementary data for this paper are available on-line only at http://jmb.or.kr.
