# Supplementary material for: Metabolome-Wide Reprogramming Modulated by Wnt/β-Catenin Signaling Pathway
Source: J Microbiol Biotechnol. 2022 Nov 18;33(1):114–22. doi: 10.4014/jmb.2211.11013 (PMC9895996; doi:10.4014/jmb.2211.11013)

Table S1. The gene information of custom qPCR array.

Position	Unigene	Symbol	Description
A01	Hs.83190	FASN	Fatty acid synthase
A02	Hs.558396	SCD	Arylacetamide deacetylase
A03	Hs.379191	SCD5	Stearoyl-CoA desaturase 5
A04	Hs.299878	DEGS1	Degenerative spermatocyte homolog 1, lipid desaturase (Drosophila)
A05	Hs.159643	DEGS2	Degenerative spermatocyte homolog 2, lipid desaturase (Drosophila)
A06	Hs.503546	FADS1	Fatty acid desaturase 1
A07	Hs.502745	FADS2	Fatty acid desaturase 2
A08	Hs.21765	FADS3	Fatty acid desaturase 3
A09	Hs.448845	FADS6	Fatty acid desaturase domain family, member 6
A10	Hs.714499	ACAA1	Acetyl-CoA acyltransferase 1
A11	Hs.200136	ACAA2	Acetyl-CoA acyltransferase 2
A12	Hs.331141	ACAD10	Acyl-CoA dehydrogenase family, member 10
B01	Hs.441378	ACAD11	Acyl-CoA dehydrogenase family, member 11
B02	Hs.567482	ACAD9	Acyl-CoA dehydrogenase family, member 9
B03	Hs.471277	ACADL	Acyl-CoA dehydrogenase, long chain
B04	Hs.445040	ACADM	Acyl-CoA dehydrogenase, C-4 to C-12 straight chain
B05	Hs.507076	ACADS	Acyl-CoA dehydrogenase, C-2 to C-3 short chain
B06	Hs.81934	ACADSB	Acyl-CoA dehydrogenase, short/branched chain
B07	Hs.437178	ACADVL	Acyl-CoA dehydrogenase, very long chain
B08	Hs.232375	ACAT1	Acetyl-CoA acetyltransferase 1
B09	Hs.571037	ACAT2	Acetyl-CoA acetyltransferase 2
B10	Hs.568046	ACOT1	Acyl-CoA thioesterase 1
B11	Hs.591756	ACOT12	Acyl-CoA thioesterase 12
B12	Hs.122038	ACOT6	Acyl-CoA thioesterase 6
C01	Hs.126137	ACOT7	Acyl-CoA thioesterase 7
C02	Hs.444776	ACOT8	Acyl-CoA thioesterase 8
C03	Hs.298885	ACOT9	Acyl-CoA thioesterase 9
C04	Hs.464137	ACOX1	Acyl-CoA oxidase 1, palmitoyl
C05	Hs.444959	ACOX2	Acyl-CoA oxidase 2, branched chain
C06	Hs.479122	ACOX3	Acyl-CoA oxidase 3, pristanoyl
C07	Hs.655760	ACSBG1	Acyl-CoA synthetase bubblegum family member 1
C08	Hs.465720	ACSBG2	Acyl-CoA synthetase bubblegum family member 2
C09	Hs.406678	ACSL1	Acyl-CoA synthetase long-chain family member 1
C10	Hs.655772	ACSL3	Acyl-CoA synthetase long-chain family member 3
C11	Hs.268785	ACSL4	Acyl-CoA synthetase long-chain family member 4
C12	Hs.11638	ACSL5	Acyl-CoA synthetase long-chain family member 5
D01	Hs.14945	ACSL6	Acyl-CoA synthetase long-chain family member 6
D02	Hs.706754	ACSM3	Acyl-CoA synthetase medium-chain family member 3
D03	Hs.450804	ACSM4	Acyl-CoA synthetase medium-chain family member 4
D04	Hs.659606	ACSM5	Acyl-CoA synthetase medium-chain family member 5
D05	Hs.604551	ALDH2	Aldehyde dehydrogenase 2 family (mitochondrial)
D06	Hs.274539	BDH1	3-hydroxybutyrate dehydrogenase, type 1
D07	Hs.124696	BDH2	3-hydroxybutyrate dehydrogenase, type 2
D08	Hs.503043	CPT1A	Carnitine palmitoyltransferase 1A (liver)
D09	Hs.439777	CPT1B	Carnitine palmitoyltransferase 1B (muscle)
D10	Hs.112195	CPT1C	Carnitine palmitoyltransferase 1C
D11	Hs.713535	CPT2	Carnitine palmitoyltransferase 2
D12	Hs.12068	CRAT	Carnitine O-acetyltransferase
E01	Hs.125039	CROT	Carnitine O-octanoyltransferase
E02	Hs.492212	DECR1	2,4-dienoyl CoA reductase 1, mitochondrial
E03	Hs.628831	DECR2	2,4-dienoyl CoA reductase 2, peroxisomal
E04	Hs.76394	ECHS1	Enoyl CoA hydratase, short chain, 1, mitochondrial
E05	Hs.15250	ECI2	Enoyl-CoA delta isomerase 2
E06	Hs.429879	EHHADH	Enoyl-CoA, hydratase/3-hydroxyacyl CoA dehydrogenase
E07	Hs.380135	FABP1	Fatty acid binding protein 1, liver
E08	Hs.282265	FABP2	Fatty acid binding protein 2, intestinal
E09	Hs.657242	FABP3	Fatty acid binding protein 3, muscle and heart (mammary-derived growth inhibitor)
E10	Hs.391561	FABP4	Fatty acid binding protein 4, adipocyte
E11	Hs.532699	GCDH	Glutaryl-CoA dehydrogenase
E12	Hs.1466	GK	Glycerol kinase
F01	Hs.98008	GK2	Glycerol kinase 2
F02	Hs.524418	GPD1	Glycerol-3-phosphate dehydrogenase 1 (soluble)
F03	Hs.512382	GPD2	Glycerol-3-phosphate dehydrogenase 2 (mitochondrial)
F04	Hs.516032	HADHA	Hydroxyacyl-CoA dehydrogenase/3-ketoacyl-CoA thiolase/enoyl-CoA hydratase (trifunctional protein), alpha subunit
F05	Hs.533444	HMGCL	3-hydroxymethyl-3-methylglutaryl-CoA lyase
F06	Hs.397729	HMGCS1	3-hydroxy-3-methylglutaryl-CoA synthase 1 (soluble)
F07	Hs.59889	HMGCS2	3-hydroxy-3-methylglutaryl-CoA synthase 2 (mitochondrial)
F08	Hs.656980	LIPE	Lipase, hormone-sensitive
F09	Hs.180878	LPL	Lipoprotein lipase
F10	Hs.94949	MCEE	Methylmalonyl CoA epimerase
F11	Hs.485527	MUT	Methylmalonyl-CoA mutase
F12	Hs.472491	OXCT2	3-oxoacid CoA transferase 2

G01	Hs.281680	PECR	Peroxisomal trans-2-enoyl-CoA reductase
G02	Hs.437403	PPA1	Pyrophosphatase (inorganic) 1
G03	Hs.43322	PRKAA1	Protein kinase, AMP-activated, alpha 1 catalytic subunit
G04	Hs.741184	PRKAB1	Protein kinase, AMP-activated, beta 1 non-catalytic subunit
G05	Hs.631630	PRKACA	Protein kinase, cAMP-dependent, catalytic, alpha
G06	Hs.487325	PRKACB	Protein kinase, cAMP-dependent, catalytic, beta
G07	Hs.530862	PRKAG1	Protein kinase, AMP-activated, gamma 1 non-catalytic subunit
G08	Hs.363138	SLC27A1	Solute carrier family 27 (fatty acid transporter), member 1
G09	Hs.11729	SLC27A2	Solute carrier family 27 (fatty acid transporter), member 2
G10	Hs.438723	SLC27A3	Solute carrier family 27 (fatty acid transporter), member 3
G11	Hs.656699	SLC27A4	Solute carrier family 27 (fatty acid transporter), member 4
G12	Hs.292177	SLC27A5	Solute carrier family 27 (fatty acid transporter), member 5
H01	Hs.49765	SLC27A6	Solute carrier family 27 (fatty acid transporter), member 6
H02	Hs.546285	RPLP0	Ribosomal protein, large, P0
H03	Hs.534255	B2M	Beta-2-microglobulin
H04	Hs.592355	GAPDH	Glyceraldehyde-3-phosphate dehydrogenase
H05	Hs.412707	HPRT1	Hypoxanthine phosphoribosyltransferase 1
H06	Hs.520640	ACTB	Actin, beta
H07	N/A	RTC	Reverse Transcription Control
H08	N/A	RTC	Reverse Transcription Control
H09	N/A	PPC	Positive PCR Control
H10	N/A	PPC	Positive PCR Control
H11	N/A	GDC	Human Genomic DNA Contamination
H12	N/A	GDC	Human Genomic DNA Contamination

Table S2. The list of significantly altered metabolites by Wnt

(Mann-Whitney U test, p-value < 0.05)

Compound	Platform	4hr-p-value	4hr-fold-change	8hr-p-value	8hr-fold-change	15hr-p-value	15hr-fold-change
(3aS,3a1S,10bR)-3a-ethyl-5-(methoxycarbonyl)-13-methyl-1,2,3,3a, 3a1,4,6,11,12,13-decahydroindolizino[8,1-cd]carbazol-13-ium chloride	LC	0.00	6.88	0.01	7.84	0.00	5.33
Alpha-D-glucose-1-phosphate	LC	0.00	3.05	0.01	2.15	0.00	1.98
Aminoadipate	LC	0.00	0.30	0.01	0.24	0.00	0.27
Byakangelicin	LC	0.00	0.15	0.01	0.04	0.00	0.08
Glutamine	LC	0.00	0.54	0.01	0.25	0.00	0.25
N-acetyl putrescine	LC	0.00	0.68	0.01	0.35	0.00	0.47
Toddalolactone	LC	0.00	6.72	0.01	4.05	0.00	8.01
3-Indoleacetic acid	LC	0.01	0.25	0.01	0.05	0.00	0.01
Oxoproline	GC	0.03	0.79	0.01	0.67	0.00	0.63
Myo-inositol	GC	0.33	0.94	0.01	0.63	0.00	0.50
4-[4-(2-Chlorophenyl)piperazin-1-yl]-4-oxobutanoic acid	LC	0.33	1.76	0.01	9.80	0.00	5.00
Guanine	GC	0.54	1.01	0.01	1.52	0.00	2.76
6-Dimethylaminopurine	LC	0.66	3.11	0.01	5.46	0.00	4.08
Arginine	LC	0.43	1.00	0.02	0.54	0.00	0.59
Galactose	GC	0.01	0.42	0.03	0.40	0.00	0.56
Glucose	GC	0.02	0.61	0.03	0.39	0.00	0.60
Tagatose	GC	0.05	0.58	0.03	0.53	0.00	0.50
Citric acid	GC	0.43	1.09	0.03	1.15	0.00	1.51
Serine	GC	0.43	0.93	0.03	0.77	0.00	0.60
Guanosine 5_-diphosphate	LC	0.43	0.54	0.03	0.21	0.00	0.19
5'-deoxy-5'-methylthioadenosine	GC	0.79	1.14	0.03	0.77	0.00	0.73
Octanoyl-L-Carnitine	LC	0.79	1.15	0.03	0.31	0.00	0.13
Argininosuccinic acid	LC	0.00	0.75	0.06	0.63	0.00	0.59
Isopentenyladenine	LC	0.02	16.78	0.06	2.35	0.00	8.82
Uracil	GC	0.03	0.82	0.06	1.30	0.00	2.13
5-Aminovaleric acid betaine	LC	0.05	0.84	0.06	0.72	0.00	0.44
N-epsilon-Acetyllysine	LC	0.05	1.77	0.06	1.66	0.00	4.09
3-phenyllactic acid	GC	0.18	1.22	0.06	1.31	0.00	2.61
Phosphorylcholine	LC	0.08	0.87	0.10	0.69	0.00	0.42
1-(Aminocarbonyl)-L-proline	LC	0.54	0.96	0.10	0.73	0.00	0.75
Securinine	LC	0.54	0.82	0.10	0.57	0.00	0.41
N6-Isopentenyladenosine	LC	0.05	2.04	0.15	1.43	0.00	1.86
Xanthine	GC	0.08	1.28	0.15	1.21	0.00	1.85
2'-Deoxyadenosine-5'-monophosphate	LC	0.13	1.44	0.15	1.25	0.00	2.11
Fructose	GC	0.25	0.81	0.15	0.79	0.00	0.49
Aspartic acid	GC	0.02	0.79	0.22	0.78	0.00	0.52
N-Acetyl-Asp-Glu	LC	0.02	0.84	0.22	0.73	0.00	0.64
Fumaric acid	GC	0.03	0.76	0.22	0.84	0.00	0.78
L-Saccharopine	LC	0.08	0.78	0.22	0.67	0.00	0.60
Mannose	GC	0.13	0.23	0.31	0.42	0.00	0.48
Glucosamine	LC	0.79	0.49	0.31	0.60	0.00	2.04
Adenosine	GC	0.18	1.33	0.42	1.10	0.00	1.69
Metribuzin-diketo	LC	0.79	1.03	0.42	0.82	0.00	0.61
Thymine	GC	0.05	0.85	0.55	1.06	0.00	1.74
O-isobutyrylcarnitine	LC	0.43	0.92	0.55	0.75	0.00	0.47
7-methoxy-2-methyl-3-phenyl-4H-chromen-4-one	LC	0.93	0.98	0.55	0.91	0.00	3.27
Ophthalmate	LC	0.05	1.24	0.69	0.81	0.00	0.46
Fructose-6-phosphate	GC	0.33	0.77	0.69	0.92	0.00	3.29
Nicotinic acid mononucleotide	LC	0.54	1.07	0.69	1.16	0.00	1.98
2`-O-Methylguanosine	LC	1.00	1.01	0.69	0.84	0.00	1.57
Guanosine 5'-monophosphate	LC	0.08	0.83	0.84	0.93	0.00	1.48
Hypoxanthine	GC	0.79	1.06	0.84	1.00	0.00	1.90
Edetic acid	LC	1.00	0.98	0.84	1.05	0.00	4.81
Inosine	GC	0.25	1.22	1.00	0.95	0.00	1.74
Isonicotinic acid	LC	0.43	0.59	1.00	1.29	0.00	1.86
Adenosine 5'-monophosphate	LC	0.66	1.04	1.00	1.01	0.00	1.42
N-acetylneuraminate	LC	0.66	0.78	0.03	0.28	0.00	0.58
Adenosine 5'-monophosphate sodium salt	LC	0.43	0.85	0.10	1.52	0.00	3.18
Beta-Nicotinamide adenine dinucleotide	LC	0.43	0.88	0.10	0.55	0.00	0.70
Glutamic acid	LC	0.54	0.93	0.10	0.64	0.00	0.75
N,N-Dimethylarginine	LC	0.00	1.90	0.22	1.35	0.00	1.64
Glutathione oxidized	LC	0.79	0.90	0.55	0.81	0.00	0.72
2-Hydroxybenzonitrile	LC	0.93	1.07	1.00	1.05	0.00	3.22
Deoxycarnitine	LC	1.00	1.02	1.00	0.96	0.00	0.64
Ergosterol	GC	0.02	1.76	0.01	1.95	0.01	1.79
5-Methylcytidine	LC	0.79	1.30	0.01	0.29	0.01	0.40
Succinic acid	GC	0.00	0.74	0.02	0.59	0.01	0.70
Cystathionine	LC	0.25	0.62	0.15	0.46	0.01	0.29
Dehydrosalsolidine	LC	0.13	1.35	0.31	0.81	0.01	0.51
Adipic acid	GC	0.13	0.79	0.42	1.07	0.01	0.76
N-Acetylarginine	LC	0.79	0.89	0.42	1.16	0.01	1.29
Taurine	LC	0.02	1.74	0.69	0.63	0.01	0.82
Creatine	LC	0.66	1.05	0.69	0.94	0.01	0.83
N-methylalanine	GC	0.54	1.25	1.00	0.97	0.01	0.64
Di-n-butyl phthalate	LC	0.13	1.83	0.01	2.90	0.02	2.21
Palmitoleic acid	GC	0.33	1.15	0.01	1.39	0.02	1.20
Palmitic acid	GC	0.66	1.01	0.01	1.23	0.02	1.26
Adenosine diphosphate ribose	LC	0.25	0.77	0.03	0.31	0.02	0.53
Propionic acid	LC	0.02	0.46	0.06	0.30	0.02	0.68
1-monopalmitin	GC	0.54	1.18	0.15	1.08	0.02	1.18
LPC 16:0	LC	0.05	1.80	0.22	1.37	0.02	0.71
Tetradecanoyl-L-Carnitine	LC	0.43	1.10	0.31	0.72	0.02	0.42
3-Methyladenine	LC	0.33	0.89	1.00	1.00	0.02	1.51
S-adenosyl-L-homocysteine	LC	0.33	0.32	1.00	0.30	0.02	0.62
Benzoic acid	GC	0.13	1.21	0.01	1.44	0.03	1.33
Heptadecanoic acid	GC	0.18	0.89	0.01	1.23	0.03	1.23
Asparagine	GC	0.79	0.89	0.01	0.78	0.03	0.67
Oleoyl Ethanolamide	LC	0.25	1.71	0.02	1.77	0.03	1.68
Guanosine	GC	0.33	1.32	0.06	1.50	0.03	1.79
Glutathione reduced	LC	0.93	1.08	0.06	0.28	0.03	0.77
LPE 16:0	LC	0.05	1.71	0.10	1.36	0.03	0.71
Threonine	GC	0.79	1.02	0.15	0.73	0.03	0.53
Pterin	LC	0.08	0.79	0.22	0.57	0.03	0.66
2-O-Methyladenosine	LC	0.54	1.36	0.22	1.53	0.03	2.45
Lysine	GC	0.93	1.02	0.22	0.87	0.03	0.78
Inosine 5'-monophosphate	GC	0.00	2.12	0.42	1.28	0.03	1.53
Pentadecanoic acid	GC	0.66	0.95	0.55	1.03	0.03	1.17
1-Methylnicotinamide	LC	0.00	1.63	0.69	1.29	0.03	1.86

L-beta-Homolysine	LC	0.66	1.09	0.84	0.98	0.03	0.56
Stearic acid	GC	0.79	1.03	0.01	1.26	0.04	1.25
N,N-Dimethyldodecylamine	LC	0.54	17.59	0.03	36.57	0.04	2.03
Laminine	LC	0.33	0.90	0.42	0.86	0.04	0.85
Methionine	GC	0.43	0.87	0.42	0.92	0.04	0.69
S-Lactoylglutathione	LC	0.66	0.22	0.42	0.02	0.04	0.00
Cordycepin	LC	0.79	1.13	0.42	1.41	0.04	0.44
Arabitol	GC	0.00	0.42	0.01	0.50	0.06	0.41
Glycerol	GC	0.43	1.06	0.01	1.22	0.06	1.22
Uridine-5'-diphosphate-glucose	LC	0.02	0.41	0.02	0.29	0.06	0.64
Lauroylcarnitine	LC	0.08	0.43	0.02	0.41	0.06	0.31
Pyrophosphate	GC	0.25	0.84	0.02	0.45	0.06	0.76
Melamine	LC	0.02	1.70	0.55	1.67	0.06	0.28
Ectoine	LC	0.02	2.09	0.84	1.20	0.06	0.89
Lactic acid	GC	0.43	0.93	0.01	0.73	0.09	1.22
Beta-Alanine	LC	0.03	1.29	0.84	0.81	0.09	0.82
Oleic acid	GC	0.93	1.02	0.01	1.23	0.13	1.14
N-alpha-Acetyl-L-lysine	LC	0.79	1.21	0.03	2.27	0.18	1.83
LPC 18:1	LC	0.03	1.93	0.01	1.71	0.24	0.88
Methylmalonate	LC	0.66	1.10	0.01	2.22	0.24	0.58
Arachidonic acid	GC	1.00	0.94	0.01	1.24	0.24	1.14
Riboflavin	LC	0.43	1.70	0.02	2.74	0.24	1.50
Adenosine_Diphosphate	LC	1.00	0.97	0.02	0.44	0.24	0.93
Lauramidopropyl betaine	LC	0.03	2.56	0.01	2.26	0.31	1.32
Homoserine	GC	0.66	1.34	0.02	1.58	0.31	1.39
N-Methylaniline	LC	1.00	0.95	0.02	1.42	0.31	1.04
Dioctyl Phthalate	LC	0.08	1.82	0.03	1.75	0.31	1.43
Tetraethylene glycol monododecyl ether	LC	0.00	2.36	0.01	5.19	0.39	1.02
Acetyl CoA	LC	0.02	4.46	0.84	1.86	0.39	1.94
Putrescine	GC	0.13	0.73	0.01	0.66	0.48	1.14
Lumichrome	LC	0.13	1.45	0.01	1.68	0.48	1.10
LPC 17:0	LC	0.03	2.10	0.69	1.31	0.48	0.71
2,2,6,6-Tetramethyl-4-piperidone	LC	0.18	1.28	0.02	2.22	0.59	0.85
2'-Deoxyadenosine	LC	0.54	1.17	0.03	1.82	0.59	0.93
Cytidine 5'-diphosphocholine	LC	0.00	0.72	0.22	0.75	0.59	1.09
Erucamide	LC	0.54	1.34	0.03	2.46	0.70	1.38
Hexonic acid	GC	0.93	0.98	0.03	1.49	0.70	0.96
Isobutyric acid	LC	0.00	2.88	0.55	0.81	0.70	1.22
Tigogenin	LC	1.00	0.96	0.01	3.55	0.82	1.10
Benzoylcholine	LC	0.66	1.30	0.03	1.77	0.82	1.16
Phthalic anhydride	LC	0.03	1.92	0.06	1.94	0.82	1.30
(2E,4E)-N-(2-methylpropyl)dodeca-2,4-dienamide	LC	0.66	1.16	0.01	4.61	1.00	0.85
3,4-Methylenedioxymethamphetamine	LC	0.66	1.20	0.01	1.44	1.00	0.96
N-Palmitoylsphingosine	LC	0.02	0.19	0.69	1.20	1.00	0.62

Figure S1. The metabolite changed according to the Wnt-activation
(A) The list of significantly changed metabolites at the time points. Common features at the all time-point are 13 metabolites. (B) The box & dot plot of glucose, glucose 1-phosphate, and galactose

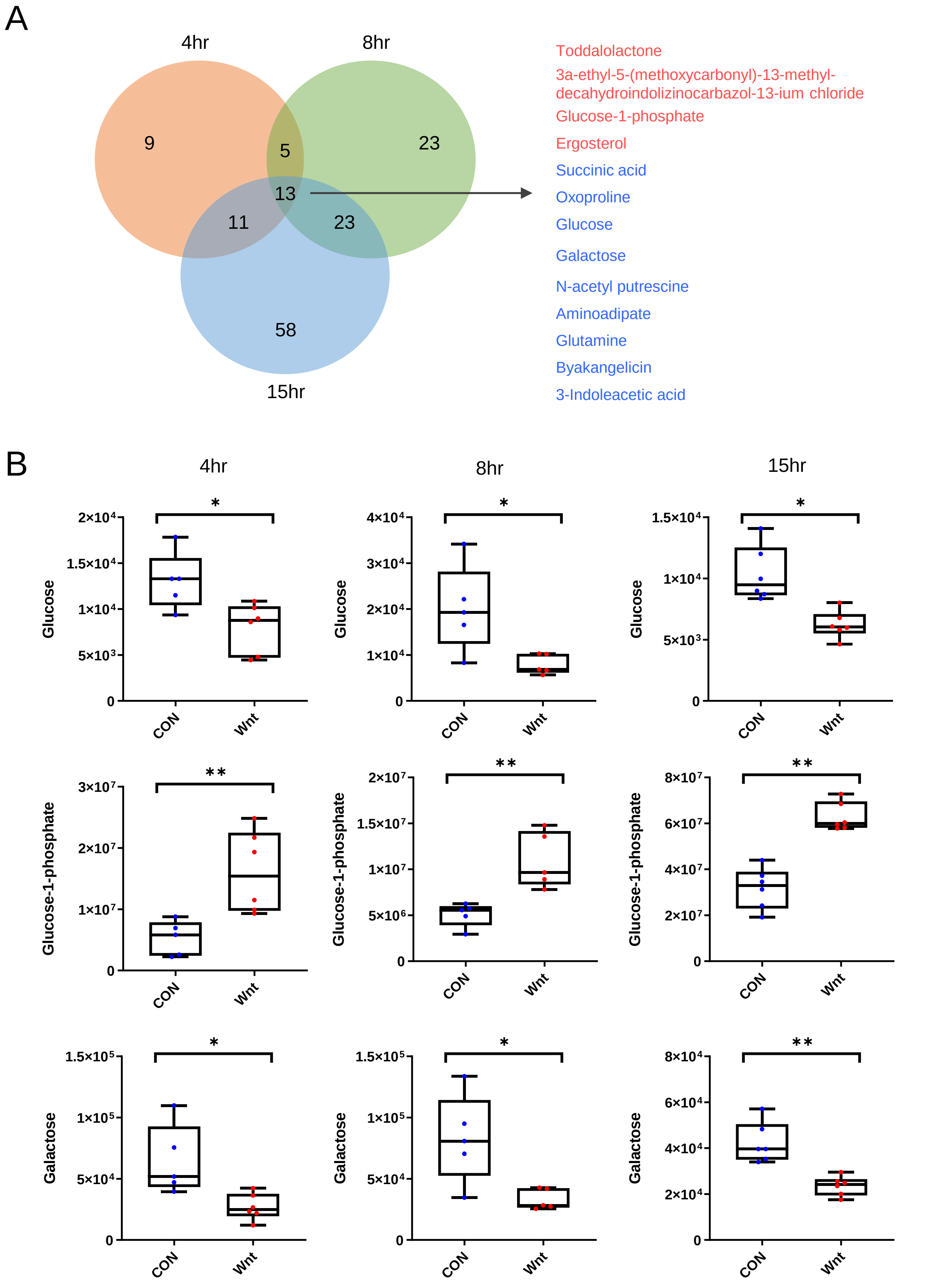

Supplement: Supplementary file 1 [file jmb-33-1-114-supple.pdf]
